# Lateral Spinal Artery Aneurysm Causing Subarachnoid Hemorrhage: Literature Review and Case Report

**DOI:** 10.3390/jcm13164910

**Published:** 2024-08-20

**Authors:** Yoo Sung Jeon, Jeong Jin Park, Young Il Chun, Hong Gee Roh

**Affiliations:** 1 Department of Neurosurgery, Konkuk University Medical Center, Seoul 05030, Republic of Korea; libix@hanmail.net (Y.S.J.); yichun@kuh.ac.kr (Y.I.C.); 2 Department of Neurology, Konkuk University Medical Center, Seoul 05030, Republic of Korea; parkjj@kuh.ac.kr; 3 Department of Radiology, Konkuk University Medical Center, Seoul 05030, Republic of Korea

**Keywords:** lateral spinal artery aneurysm, subarachnoid hemorrhage, cerebral angiography, posterior spinal artery

## Abstract

Ruptured aneurysms of the lateral spinal artery (LSA) causing subarachnoid hemorrhage (SAH) are exceptionally rare. Unlike common aneurysms in the circle of Willis, LSA aneurysms present unique diagnostic and therapeutic challenges due to their complex anatomy. We reviewed the literature, examining case reports detailing LSA aneurysm occurrences, diagnoses, treatments, and complications, and our subsequent analysis highlights the clinical presentations, imaging findings, treatment methods, and anatomical features of the LSA. We identified 10 patients from 7 case reports of LSA aneurysm presenting with SAH, and combined with the present case, this comprised a total of 11 patients. An initial CT angiography identified LSA aneurysm in only 2 of 11 patients, while 5 cases were detected in transfemoral cerebral angiography. Seven patients had stenosis or occlusion of nearby arteries. Among the 10 patients treated, 7 underwent microsurgical clipping, and 3 had endovascular treatment; complications included PICA infarction and subdural hematoma. LSA aneurysms, though rare, should be considered in differential diagnoses of posterior fossa SAH. An accurate diagnosis often requires repeated imaging. It is proposed to individualize treatment strategies based on the unique anatomical structure and hemodynamic conditions of each patient, utilizing both endovascular and surgical approaches. Understanding the vascular anatomy and collateral pathways of the LSA is crucial for improving diagnostic accuracy and treatment outcomes.

## 1. Introduction

The lateral spinal artery (LSA) is a critical component of the spinal vascular network, bridging extradural and intradural blood supplies. It originates from the intradural segment of the vertebral artery (VA) at the C1 level and travels caudally and laterally, aligning with the pathway of the spinal component of the 11th cranial nerve [1,2]. The LSA is strategically positioned between the dentate ligament and the posterior spinal nerve roots, and it extends its influence through connections with the posterior inferior cerebellar artery (PICA) and posterior radicular branches, particularly at the C2 level [3,4,5,6,7]. Additionally, the LSA contributes to the vascular architecture by forming significant pial anastomoses with the contralateral system and developing posterior medullary superficial arteries in the lower medulla oblongata [1,2]. Importantly, it forms anastomoses with the posterior spinal artery (PSA) around the C4 or C5 level, which are crucial for its role in supporting the neurovascular integrity of the spinal and cerebellar regions [3,5,6,7].

Clinically, aneurysms involving the LSA are rare but noteworthy due to their potential to present with severe complications such as subarachnoid hemorrhage (SAH), which accounts for approximately 1% of all SAH cases [2,5,8]. The complexity of these aneurysms is highlighted by their associations with conditions such as arteriovenous malformations (AVMs), spinal dural arteriovenous fistulas (AVFs), and aortic coarctation, making isolated saccular spinal artery aneurysms extremely rare and clinically significant [1,5,9,10,11].

The limited number of reported LSA aneurysm cases, combined with the LSA’s complex anatomical structure, poses significant challenges in achieving accurate diagnoses and determining effective treatment strategies [5]. To address these challenges, we aim to synthesize various reports to enhance the anatomical understanding of the LSA, devise methods to improve diagnostic accuracy, and propose imaging techniques for evaluating patients with posterior fossa SAH based on the recent literature. Utilizing the precise anatomical information obtained, we seek to predict and manage potential postoperative complications and side effects, thereby improving outcomes through the selection of appropriate treatment methods.

This article aims to explore the complex vascular role of the LSA and discusses the surgical and therapeutic strategies that arise from the intricate interplay of anatomy and pathology in this critical area of the craniocervical junction. Through this exploration, we aim to enhance the understanding of and improve outcomes for conditions associated with the LSA.

## 2. Method

### 2.1. Literature Search and Selection

This review article systematically examined the existing research on SAH resulting from LSA aneurysm rupture. We conducted a comprehensive search using databases such as PubMed, Google Scholar, and Web of Science. The search terms included “lateral spinal artery aneurysm”, “SAH”, “LSA aneurysm SAH”, and “cervico-cranial junction vascular anatomy”. We focused on articles published within the last 25 years to ensure that the review covers the most recent and relevant studies.

### 2.2. Inclusion and Exclusion Criteria

#### 2.2.1. Inclusion Criteria

This study included original articles, literature reviews, and case reports discussing LSA aneurysm and associated SAH, providing detailed anatomical or clinical insights.

The diagnostic method was confirmation via transfemoral cerebral angiography (TFCA), with no discrepancies between LSA and PSA.

#### 2.2.2. Exclusion Criteria

The exclusion criteria were as follows: dural AVF, AVM, anterior spinal artery aneurysm, posterior spinal artery aneurysm at the cervico-cranial junction, and others (e.g., extracranial PICA and radiculomedullary artery).

### 2.3. Data Extraction and Analysis

Data extraction from the selected case reports was performed systematically. Key information such as the clinical presentation, diagnostic methods, treatment options, and outcomes of lateral spinal artery aneurysms with SAH was collected.

## 3. Results

References for this review were found according to the inclusion/exclusion criteria. There were some cases of SAH caused by ASA, PSA, radiculomedullary AVM, and dural AVF at the cervico-cranial junction, but there were only seven references in total related to LSA aneurysm rupture. This included six case reports and one literature review with a case series, comprising a total of 10 patients.

### 3.1. Case Series

The patients’ clinical condition, the location of the aneurysm and the surrounding structures, the treatment methods and timing of diagnosis, and any complications are described in Table 1.

Briefly, there were four male (36.4%) and seven female (63.6%) patients, with a median age of 64 years (range 49~78 years old). Except for one patient who was transferred to another hospital, all others received treatment and were followed up. Of the 11 patients, 10 had Hunt and Hess grade (H-H grade) 1 to 3, and 1 had H-H grade 4. Excluding 1 patient who was lost to follow-up, 9 out of 10 patients (90%) had good outcomes, and the remaining patient deteriorated due to a different cause.

In the case series, only two cases (No. 6 and No. 11) were suspected to have LSA aneurysms based on the initial computed tomography (CT) angiography upon admission. In addition, in five cases (No. 1, No. 4, No. 5, No. 8, and No. 9), the LSA aneurysm was detected during the first TFCA. Thus, in seven cases, the LSA aneurysm was promptly identified, and in two cases (No. 8 and No. 9), delayed microsurgical clipping was performed after the discontinuation of antiplatelet medication.

In two cases (No. 2 and No. 7), the LSA aneurysm was detected during the second TFCA, and in one case (No. 3), it was discovered during the fourth TFCA. The last case (No. 10) was identified after three CT angiography and two TFCA examinations.

Among the 11 cases, vascular abnormalities were observed in 7 of them. Six cases showed severe stenosis or occlusion of the VA, with enlargement of the LSA, through which the PICA was reconstituted. In the present case, although there was VA aplasia, the ipsilateral PICA was supplied by the contralateral VA, with no direct connection between the LSA and PICA. In the remaining four cases (No. 4, No. 7, No. 8, and No. 10), no direct connection between the PICA and LSA was observed.

Including this case, a total of 10 out of 11 cases were treated by microsurgical clipping (7 cases) or endovascular treatment (3 cases). One case (No. 10) was transferred to another hospital and was not followed up. Among the clipping cases, five cases used the suboccipital craniotomy approach, one case used the far lateral transcondylar approach (No. 7), and the approach for the remaining case was not described (No. 3). In addition, three cases (No. 6, No. 7, and No. 8) additionally underwent C1 hemilaminectomy, and patient No. 6 underwent vascular anastomosis between the LSA and PICA. In the case of No. 4, the LSA aneurysm was not observed during the first clipping attempt but was successfully treated in the second attempt. Among the three cases treated with endovascular treatment, internal trapping was performed in two patients (No. 1 and No. 5), including the aneurysm, while the present case involved proximal occlusion.

Postoperative complications occurred in a total of four cases. PICA infarction occurred in two cases where internal trapping was performed via the endovascular approach. Among these, patient No. 1 required cerebellar decompression, and in the microsurgical group, patient No. 2 developed a postoperative subdural hematoma and underwent burr hole trephination. Furthermore, two patients (No. 2 and No. 7) required external ventricular drainage due to acute hydrocephalus, and two patients developed chronic hydrocephalus and received a ventriculoperitoneal shunt (No. 3) and a lumboperitoneal shunt (No. 5).

Regarding clinical outcomes, six patients had no neurologic deficit, while four patients experienced deficiencies such as hemiplegia. Three patients showed improvement during follow-up, and one case (No. 2) resulted in death due to acute embolic occlusion of the superior mesenteric artery, which was unrelated to the treatment.

### 3.2. Case Description

#### 3.2.1. Clinical Presentation

A 51-year-old woman experienced a sudden onset of severe headache with posterior neck pain. Her past medical history was notable for chronic alcoholism and hepatitis. On admission, she presented with mild drowsiness but no overt neurological deficits. An immediate brain CT scan was performed, revealing a diffuse subarachnoid hemorrhage in the posterior cranial fossa.

#### 3.2.2. Radiological Findings

**Figure 1:**
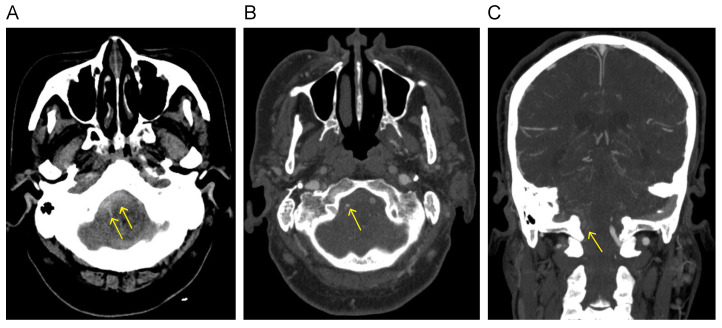
(**A**) Initial CT scan displays SAH (arrow) in the posterior fossa and around the midbrain. CT angiography reveals a tiny suspected aneurysm (arrow) at the right cerebellomedullary cistern in axial (**B**) and coronal views (**C**).

The initial CT scan displayed acute SAH in the posterior fossa and around the midbrain, accompanied by a small intraventricular hemorrhage (IVH) (Figure 1A). There was also evidence of mild hydrocephalus. CT angiography revealed aplasia of the right V4 segment of the VA, which terminated as the posterior meningeal artery (PMA). Additionally, a fine irregular vessel with a tiny suspected aneurysm measuring 1.5 mm was identified at the right cerebellomedullary cistern (Figure 1B,C).

**Figure 2:**
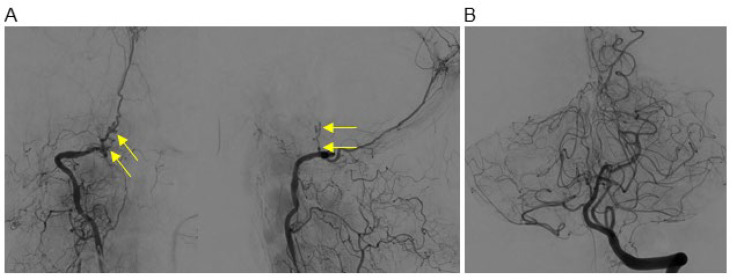
(**A**) Cerebral angiography reveals a small irregular branch exhibiting multifocal severe stenoses and dilatations (arrow) in routine AP and lateral views. (**B**) Bilateral PICAs originate from the contralateral VA.

The cerebral angiography results revealed that the right VA terminated as the PMA. Just before its connection to the PMA, a small irregular branch was identified, exhibiting multifocal severe stenoses and dilatations (Figure 2A). The terminal portion of this irregular branch contained a relatively large aneurysm with partial contrast filling, suspected to be the ruptured aneurysm based on three-dimensional rotational angiography (3D-RA) (Video S1). Additionally, bilateral PICAs originated from the contralateral VA (Figure 2B).

#### 3.2.3. Endovascular Treatment

**Figure 3:**
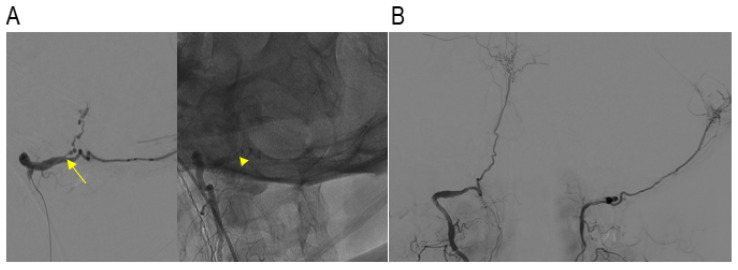
(**A**) The microcatheter (arrow) was carefully advanced to the proximal dissection point, and a coil was cautiously inserted (arrowhead), focusing on the proximal dissection point in oblique view. (**B**) Delayed angiograms showed progressive flow reduction, and the final angiogram confirmed no flow through the dissection segment at the coiling site.

We elected to treat this patient via an endovascular approach. A 6F sheath (Terumo, Tokyo, Japan) was placed, and a 6F Envoy guiding catheter (Codman Neurovascular, Raynham, MA, USA) was positioned in the right V2 segment of the VA. A Synchro2^®^ guidewire (Stryker Neurovascular, Cork, Ireland) was used to navigate an Excelsior SL-10 microcatheter (Stryker Neurovascular) to the distal V3 segment of the VA, followed by selective angiography. The dissection segment appeared long and irregular. Given the impossibility and high risk of advancing the microcatheter to the aneurysm, a decision was made to proceed with proximal occlusion. The microcatheter was carefully advanced to the proximal dissection point, and coiling was successfully performed using a Target Helical NANO (1 mm/1 cm) coil (Stryker Neurovascular) (Figure 3A). A control angiography revealed residual contrast filling in the dissection segment and aneurysm, although the blood flow was significantly reduced. Five-minute and ten-minute delayed angiograms showed progressive flow reduction and slowdown. The final angiogram confirmed the absence of flow through the dissection segment at the coiling site (Figure 3B).

#### 3.2.4. Postoperative Course

Magnetic resonance imaging (MRI) performed four days after the endovascular procedure revealed no infarcts in the PICA territory or brainstem. TFCA performed at one week showed complete occlusion of the lateral spinal artery aneurysm. The patient’s consciousness was clear, and her headache gradually improved without any neurological deficits. On the 14th day, she was discharged from the hospital due to an improved SAH resolution and hydrocephalus on the initial CT. At the 12-month follow-up, MRI and magnetic resonance angiography showed no further infarctions or de novo aneurysms.

### 3.3. Relationship between PICA, LSA, and PSA

The LSA originates from either the PICA or the intradural VA. It descends parallel to the spinal component of the 11th cranial nerve, posterior to the dentate ligament, and anterior to the posterior spinal nerve roots up to the C4 level [3,14]. The LSA supplies the lateral and posterior surfaces of the spinal cord and anastomoses with the PICA and other regional arteries [1,3].

**Figure 4:**
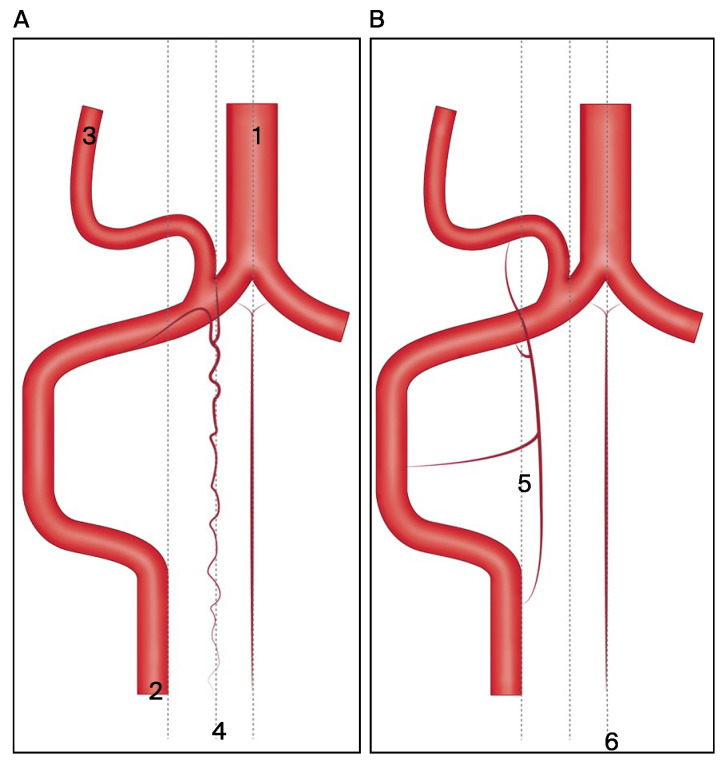
The classification was simplified based on the relationship between the PICA, LSA, and PSA. (**A**) Origin and branching patterns of PICA and PSA: PICA originating from V4 segment of VA and PSA splitting into descending posterolateral and ascending branches merging with PICA. (**B**) Segmental connections and cranial anastomosis of LSA: LSA is the outermost cervical spinal artery connecting with VA from C1 to C4 and anastomosing with PICA. Abbreviations: 1 = basilar artery; 2 = vertebral artery; 3 = posterior inferior cerebellar artery; 4 = posterior spinal artery; 5 = lateral spinal artery; 6 = anterior spinal artery.

The classification was simplified based on the relationship between the PICA, LSA, and PSA (Figure 4). The PICA usually originates from the V4 segment of the VA. The PSA arises from the extradural segment of the VA and runs parallel to it, and then it splits into two branches: a descending posterolateral branch and an ascending branch that merges with the PICA (Figure 4A). The LSA is the outermost of the three cervical spinal arteries. It forms multiple segmental connections with the VA from C1 to C4, and it also creates a cranial anastomosis with the PICA (Figure 4B).

## 4. Discussion

The LSA plays a crucial role in the spinal vascular system, yet its precise definition and function often provoke debate due to its complex interactions with other spinal and cerebral arteries. Typically emerging from the intradural segment of the VA at the C1 level, the LSA travels laterally around the spinal cord and converges with the PSA near the C4 and C5 vertebrae [1,2]. It then rises to form an anastomosis with the PICA, which is essential for supplying blood to the medulla [1,15]. Despite its critical role in vascularizing the spine and medulla, the LSA’s small size and complex anatomy make it difficult to clearly visualize on imaging studies like CT or MR angiography.

Due to various unknown causes, the occurrence of a lateral spinal artery aneurysm within the complex vascular structure can lead to intracranial hemorrhage, such as SAH, at the craniocervical junction. In this case series of seven patients, stenosis or occlusion of the VA or PICA was observed around the LSA aneurysm. In addition, in some cases of V4 VA occlusion or severe stenosis, the distal V4 VA was being reconstituted through the LSA or PICA, or the PICA was being formed through the LSA [5,6,8,12]. This indicates that the LSA is integrated into a collateral system with adjacent vascular structures. Therefore, this finding suggests that stenosis or occlusion of the primary vessel may impose hemodynamic stress on the collateral vessels, potentially contributing to the development of vascular complications such as aneurysms [6,7,8]. However, in four cases, no distinct angiographic connection between the PICA and LSA was observed, and they originated independently, suggesting that other causes such as dissection should also be considered.

In this case series, two cases (18.2%) were suspected to have lateral spinal artery aneurysms based on CT angiography immediately upon admission [8]. Additionally, in five cases (45.5%), LSA aneurysm was not identified on the initial CT angiography but was detected on TFCA [5,7,10,12]. Therefore, seven patients (63.6%) had LSA aneurysms detected very quickly after the onset of SAH. Another two cases were found on the second TFCA, one case was found on the fourth TFCA, and the remaining one was identified after three CT angiography and two TFCA examinations (1 week~1 month) [5,6,10,13]. Repeated imaging over time increases the likelihood of identifying these aneurysms, which are often missed on the first scan due to their fine vascular structure and the potential presence of thrombus within the aneurysm obscuring the contrast [10,16,17]. In addition, even when LSA aneurysms are promptly detected, it may be difficult to clearly distinguish their relationship with the surrounding complex vascular structures using CT angiography or TFCA. In such cases, cerebral angiography confirmed the LSA aneurysm, particularly through the use of a superselective injection of the parent vessel [6].

Additionally, in the case series, there were no instances of this specific examination method, but Hiramatsu et al. analyzed the LSA anatomy using contrast-enhanced cone-beam CT (CE-CBCT) to study craniocervical junction AVF [18]. They performed CE-CBCT imaging after obtaining 3D-RA by infusing a 20% iodine contrast medium with a concentration of 300 mg/mL at a flow rate of 2.0 mL/s, with a 4.0 s delay and a total volume of 48 mL. Although it might have been easier to identify the structure if the LSA feeders in AVFs were more likely to be enlarged compared to LSA aneurysms, their method (CE-CBCT) was definitely superior to other imaging techniques [18]. Additionally, Tomoyuki Tsumoto et al. reported that high-resolution CBCT helps in identifying the shunt point of dural AVF [19]. T. Hiu et al. referred to the examination using the CBCT system as DynaCT and stated that it aids in the reliable visualization of small vessels and fine osseous structures, particularly in the diagnosis of dural AVF [20]. Furthermore, T.D. Aadland et al. noted that, in the case of spinal dural AVF, CBCT angiography improves visualization for precise anatomic characterization [21]. Additionally, arterial spin labeling (ASL) MRI could be helpful in situations where it is challenging to clearly evaluate the underlying causes due to fine vessels and complex collateral systems. Matteo De Simone et al. discussed the usefulness and role of ASL MRI in cerebral AVM [22]. They reported that ASL MRI, being noninvasive and having fewer complications compared to cerebral angiography, is beneficial for an acute diagnosis and functional assessment in shunt diseases. Therefore, persistent clinical suspicion and follow-up imaging through various diagnostic methods are crucial for the accurate diagnosis of LSA aneurysms in patients who do not show clear evidence of aneurysms on their initial scans. Emerging noninvasive imaging techniques like ASL MRI could be helpful in distinguishing the presence of shunt diseases in complex structures such as AVM or dural AVF. Specifically, a superselective injection of the parent vessel through cerebral angiography is thought to be highly beneficial for accurate diagnosis, and although further research is needed, CE-CBCT can also be helpful.

The management of LSA aneurysms encompasses three primary modalities: endovascular coil embolization, microsurgical clipping, and conservative treatment. Conservative management may be considered for patients with poor overall health; however, proactive intervention is typically advised to mitigate the risk of re-rupture [10]. The endovascular approach is often preferred for aneurysms in the posterior circulation due to its minimally invasive nature and association with favorable outcomes [23]. Additionally, if the parent vessel is intact and there is sufficient collateral circulation, the likelihood of infarction is reduced [4]. However, this method can be particularly challenging in the case of LSA aneurysms due to the tortuous anatomy and very small diameter of the LSA, especially when there is stenosis or occlusion of the parent artery [5]. These anatomical complexities can impede the successful navigation and deployment of endovascular devices. When endovascular access is not feasible due to these anatomical constraints, surgical clipping via suboccipital craniectomy becomes a viable alternative [5,6,10,12]. However, even though it facilitates direct access and secure aneurysm exclusion, it carries the potential for increased procedural morbidity, and sparing the very small collateral vessels and protecting cranial nerves can be challenging [5,10,23].

In our case, although the lateral spinal artery had a highly tortuous course and a very small vessel diameter, we decided to attempt proximal occlusion. This decision was based on the anatomical configuration where bilateral PICAs originated from the contralateral vertebral artery, and the ipsilateral VA terminated as the PMA. Given this unique vascular arrangement, where no direct connection between the LSA and PICA was observed, we thought that the likelihood of brainstem infarction was low, even if proximal occlusion was performed. Consequently, we performed proximal occlusion of the LSA, achieving complete occlusion of the ruptured aneurysm without occurrences of brainstem infarction or postoperative complications. However, as observed in patients No. 1 and No. 5 in the case series, when the LSA has direct connections with the PICA, distal VA, or BA, performing internal trapping can lead to PICA infarction [5,7]. This suggests that complex micro-collaterals may exist between the VA, PICA, LSA, and PSA, indicating that, if the LSA is the primary blood supplier, internal trapping could lead to brainstem ischemia, even if retrograde filing is present. This risk highlights the importance of carefully evaluating the vascular anatomy before deciding on treatment, especially in cases where the PICA or distal VA is reconstituted through very small LSA or PSA vessels [8].

In conclusion, the lateral spinal artery’s complex anatomy and integration into the spinal and cerebral vascular systems make it a challenging but critical vessel to understand and manage, particularly in cases of aneurysm. Persistent clinical suspicion, advanced imaging techniques, and a thorough understanding of the patient’s vascular anatomy are essential for an accurate diagnosis and effective treatment. The decision between endovascular and surgical approaches should be carefully considered based on the specific anatomical features and the potential risks of each method. Further research utilizing techniques such as 3D-RA and CE-CBCT may provide additional insights and improve the management of these complex cases [18,19,20,21]. Generally, LSA aneurysms are extremely rare, while dural AVFs or arteriovenous malformations are relatively more common. Therefore, further analysis of and research on these conditions could lead to the development of better diagnostic methods for LSA aneurysms and related vascular abnormalities.

## 5. Limitation

This study has several limitations. First, LSA aneurysms are extremely rare, resulting in a limited number of cases, making it difficult to generalize the findings. Second, this study is based on a retrospective literature review and case reports, lacking a systematic analysis comparing various treatment methods and long-term outcomes. Lastly, due to the variability in patients’ anatomical structures and hemodynamic conditions, the selection and outcomes of treatment methods can differ, making it challenging to establish standardized treatment protocols.

## 6. Conclusions

LSA aneurysms are rare and pose significant diagnostic challenges due to their complex anatomical relationships. In instances of SAH in the posterior fossa, while non-aneurysmal SAH remains a possibility, it is imperative to consider and investigate other potential causes through repeated and meticulous imaging studies. A comprehensive evaluation of the collateral circulation with surrounding vessels is essential before selecting the appropriate treatment modality. This thorough approach is crucial for minimizing surgical complications and achieving optimal patient outcomes.

## Figures and Tables

**Table 1:** Summary of patients with LSA aneurysms [5,6,7,8,10,12,13].

Patient Number (No.)	Age/Sex	H-H Grade ^a^	Location of Aneurysm	Other Vascular Disease at VBJ	Time of Treatment	Treatment for Ruptured LSA An.	Complications Related to Treatment
1. Chen et al. [5]	72/F	3	Rt. LSA	Bilat. VA stenosis	Promptly	Coil embolization Internal trapping (LSA)	PICA infarction
2. Chen et al. [5]	69/F	3	Lt. LSA	Lt. VA stenosis	One month later	Endovascular treatment failed Microsurgical clipping	Subdural hematoma
3. Kubota et al. [6]	59/F	NA	Rt. LSA	Rt. PICA occlusion	25 days after	Surgical aneurysmal resection	Disturbed consciousness
4. Kurita et al. [12]	61/M	1	Rt. LSA	None	1st: on the day of adm 2nd: 3 days later	Microsurgical clipping (twice)	None
5. Morigaki et al. [7]	78/F	4	Lt. LSA	Bilat. VA occlusion	Promptly	Coil embolization Internal trapping (LSA)	PICA infarction
6. Germans et al. [8]	49/M	2	Lt. LSA	Lt. PICA occlusion	Promptly	Microsurgical clipping + anastomosis (LSA and PICA)	None
7. Papadimitriou K et al. [13]	74/F	3	Rt. LSA	None	2 weeks later	Microsurgical clipping	None
8. Song, Y et al. [10]	55/M	2	Lt. LSA	None	2 weeks later	Microsurgical clipping	None
9. Song, Y et al. [10]	64/M	1	Lt. LSA	Bilat. VA stenosis	1 week later	Microsurgical clipping	None
10. Song, Y et al. [10]	73/F	1	Rt. LSA	None	19 days later	Transfer	Transfer
11. Present case	51/F	2	Rt. LSA	Rt. V4 aplasia	On the day of adm	Proximal occlusion (LSA)	None

Abbreviations: H-H grade = Hunt and Hess grade; VBJ = vertebrobasilar junction; VA = vertebral artery; PICA = posterior inferior cerebellar artery; LSA = lateral spinal artery; Bilat = bilateral; Rt = right; Lt = left; An = aneurysm; NA = Not Applicable; adm = admission. ^a^ Hunt and Hess grade: 0 = unruptured; 1 = asymptomatic or minimal headache; 2 = nuchal rigidity, cranial nerve palsy, moderate–severe headache; 3 = drowsiness, mild focal deficit, lethargy; 4 = stupor, severe hemiparesis, vegetative disturbance.

## Data Availability

The datasets generated and/or analyzed during the current study are available from the corresponding author on reasonable request.

## Supplementary Materials

The following supporting information can be downloaded at: https://www.mdpi.com/article/10.3390/jcm13164910/s1, Video S1: Three-dimensional cerebral angiography showing a ruptured lateral spinal artery aneurysm.

## Abbreviations

LSA	lateral spinal artery
VA	vertebral artery
PICA	posterior inferior cerebellar artery
ASA	anterior spinal artery
PSA	posterior spinal artery
PMA	posterior meningeal artery
SAH	subarachnoid hemorrhage
ICH	intracerebral hemorrhage
IVH	intraventricular hemorrhage
AVF	arteriovenous fistula
AVM	arteriovenous malformation
CT	computed tomography
MRI	magnetic resonance imaging
